# Under one roof: identification, evaluation, and treatment of chronic hepatitis C in addiction care

**DOI:** 10.1186/s13722-018-0111-7

**Published:** 2018-04-25

**Authors:** Stephen A. Martin, Jordon Bosse, Amanda Wilson, Phyllis Losikoff, Lisa Chiodo

**Affiliations:** 1 Department of Family Medicine and Community Health, University of Massachusetts Medical School, 55 Lake Avenue North, Worcester, MA 01655 USA; 2 Barre Family Health Center, 151 Worcester Road, Barre, MA 01005 USA; 3 CleanSlate Research and Education Foundation, 1 Roundhouse Plaza, Northampton, MA 01060 USA; 4 University of Massachusetts College of Nursing, 651 North Pleasant Street, Amherst, MA 01003 USA; 5 Division of Pediatric Infectious Disease, The Warren Alpert Medical School of Brown University, Box G-A1, Providence, RI 02912 USA; 6 CleanSlate Centers, 92 Grape Street, New Bedford, MA 02740 USA

**Keywords:** Opioid use disorder, Chronic hepatitis C, Cirrhosis, Hepatitis C epidemiology, Hepatitis C treatment, Treatment cascade, Project ECHO, Continuity of care

## Abstract

For over a decade, the vast majority of new hepatitis C virus (HCV) infections have been among young people who inject drugs (PWID). Well-characterized gaps in chronic HCV diagnosis, evaluation, and treatment have resulted in fewer than 5% of PWID receiving HCV treatment. While interferon-based treatment may have intentionally been foregone during part of this time in anticipation of improved oral therapies, the overall pattern points to deficiencies and treatment exclusions in the health care system. Treatment for HCV with all-oral, highly effective direct-acting antiviral medication for 12 weeks or less is now the standard of care, putting renewed focus on effective delivery of care. We describe here both the need for and process of chronic HCV care under the roof of addiction medicine.

## Background

Two profound changes in chronic hepatitis C virus (HCV) infection have occurred over the past decade. The first is the availability of medications that allow an all-oral highly effective cure. These direct-acting antiviral (DAA) agents have few adverse effects and enable treatment completion in as little as 8–12 weeks, with success rates exceeding 90% [1]. They are sorely needed given that HCV is now the leading cause of infectious death in the United States [2] and the most common cause of liver transplant [3].

The second change is the development of a bimodal chronic HCV demographic, with American Baby Boomers joined by young people who inject drugs (PWID). Among new acute HCV cases in 2014, more than two-thirds (68.2%) reported injection drug use [4]. HCV incidence had been in decline until early 2000s, a success enabled by identification of the virus in 1989. Subsequent blood product screening prior to transfusion, begun in 1990, virtually eliminated this mode of HCV transmission by 1992. Needle exchange efforts and a decrease in injection drug use further helped HCV incidence to decline to below 1 per 100,000 by the early 2000s. Between 2010 and 2015 this trend reversed, with acute HCV infections rising nearly threefold—a figure likely underestimated by a factor of 15 [2, 5].

Patients who are not able to obtain HCV treatment risk transmitting the virus to others, which in turn, leads to higher incidence of disease and subsequent illness. Doubling the number of patients treated each year, however, could decrease HCV prevalence to fewer than 100,000 cases by 2030 [6]. This approach, similar to that pursued for HIV, has been termed treatment (or cure) as prevention (CasP) [7–9]. Given the impact of HCV on individuals who are infected and their families, the health care system, and larger society, minimizing barriers and maximizing access to more easily tolerated evidence-based treatment for younger persons who inject drugs is imperative. Yet PWID are often relatively isolated, even stigmatized, by standard health care [10].

The HCV treatment cascade (see Table 1) describes the clinical course, from initially evaluating HCV risk to finally achieving sustained virologic response (SVR). There are substantial impediments at each stage [11, 12]. Two recent cohort studies examined this younger population—people with opioid use disorder (OUD) in medication-assisted treatment who have chronic HCV. Both found < 3% of patients initiated HCV treatment [13, 14].

**Table 1:** Mechanisms to address gaps in treatment cascade [16, 23]

Cascade step	Outpatient addiction treatment site advantages	Cascade step eliminated
1. Patients are diagnosed and aware of their infection	Standardized screening of all patients and counseling with results	
2. Linkage to care (Access to outpatient care)	HCV care remains at the site	✓
3. Confirmatory testing for HCV RNA	Standardized reflex testing	✓
4. Liver disease evaluation, including liver biopsy (Note: Liver biopsy is not required for all patients)	Blood-based biomarker testing, a recommended alternative to liver biopsy, can be readily performed on site	✓
5. Prescribed HCV treatment	Prescribed on site with coordination from specialty pharmacies	✓
6. Achieved a sustained virologic response (SVR), also referred to as being cured	SVR testing done on site	✓

Many barriers to treatment can be removed through treatment at a trusted place where HCV testing occurs. This would allow for immediate counseling and treatment evaluation after a positive result [15]. Given the high risk for HCV with the use of injection drugs, sites for outpatient treatment of OUD are a logical starting point to provide this range of HCV services. In 2017, this approach was strongly supported by the American Society of Addiction Medicine, which stated that “integration of service delivery, addressing the unique needs of addiction patients including HCV treatment, is strongly encouraged” [16]. HCV treatment in settings of OUD care is successful in Australia [17], Switzerland [18], and Italy [19]. Recently this success was repeated in Connecticut in a primary care setting embedded in an opioid treatment center [20].

## HCV and medication assisted treatment care integration

Over the past year, we have been learning from the pilot provision of HCV treatment as part of meeting our patients’ health needs—independent of primary care and eliminating multiple steps of the usual HCV treatment cascade (Table 1). CleanSlate Addiction Treatment Centers offers dedicated treatment for opioid and alcohol use disorders in eight states, with clinicians that include social workers, advanced practice clinicians, and physicians. Patients in treatment for OUD at CleanSlate have a median age of 36 years old, with 26% under the age of 30 and 64% under the age of 40. As such, they represent the demographic profile of the younger person with OUD and an elevated risk of chronic HCV infection. As the treatment cascade makes clear, caring for a group of patients with a high burden of chronic HCV does not inevitably lead to its cure; the combination of an effective treatment strategy together with longitudinal care is especially helpful. The average length of treatment in our buprenorphine program is over one year (SD = 1.3, range 0.2–5.5). This timeframe is well beyond the 12 weeks of HCV treatment needed and allows providers more time with their patients to develop therapeutic relationships. Additional time spent with patients allows providers to assess patients’ stability and motivation for treatment and provide education, which, in the context of the therapeutic relationship, can maximize adherence to treatment.

All patients who initiate substance use disorder treatment at CleanSlate are screened for blood-borne pathogens: Hepatitis B (HBV), Human Immunodeficiency Virus (HIV), and HCV testing that reflexes to a HCV viral count for all HCV antibody-positive specimens. By screening all patients we have found a 25–30% prevalence of positive HCV antibody; 75% of these patients with positive HCV antibody are subsequently found to have chronic HCV. All addiction providers are trained in HCV pre- and post-test counseling, including educating patients about DAA. Standard of care has been to refer all HCV infected patients to community providers for further evaluation and treatment [21].

In 2016, we began a pilot program in one of our clinics offering treatment for chronic HCV infection together with addiction treatment. Results of our pilot found that co-location of HCV treatment in our addiction clinic reduces the care cascade attrition found in PWID populations. In our pilot site sample, of the approximately 740 patients who were screened, 167 (22.5%) patients were found to have chronic HCV. All are in the process of being offered treatment. As of September 1, 2017, 72 (43%) entered DAA treatment. Among those who entered treatment, 72% received HCV treatment at CleanSlate and 28% chose to receive care via community treatment. Of the patients receiving care at our site who have reached 12 weeks after treatment completion (N = 48), 4 have been lost to follow-up and 2 did not complete treatment. One patient had a treatment failure at 12 weeks post-treatment. Our on-site rate of cure is 85% (41 of 48) using intention-to-treat analysis and 98% (41 of 42) among those for whom we have SVR data. In a similar CleanSlate without co-located care site, only 10% entered DAA care in the community.

Three HCV treatment model strategies have been critical for success; each lends itself to scaling and sustainability. First, we have in-house expertise of an infectious disease specialist with interest in both addiction care and HCV. This expert mentors advanced practice clinicians to independently treat HCV and also directs clinical care for patients with different severities of cirrhosis and associated complications. This expertise can be remotely offered to other sites. Though many sites for OUD treatment do not have infectious disease specialists, our sense is that partnerships with them allow a novel, effective way to meet the needs of a high-risk population and, by extension, contribute to cure as prevention.

Second, just as our clinical experience with OUD registries lent itself to HCV treatment, we have repurposed staff expertise with prior authorizations, medication management, and treatment adherence. Laboratory staff have been especially helpful in facilitating both our screening and treatment protocols.

Third, we are using the model of a learning, capacity-building organization, with clinicians (advanced practice clinicians and physicians) taking part in an HCV Project ECHO and developing new skills in HCV care [22]. Over the course of several months of ECHO participation—including presenting de-identified cases and listening to others’ cases—we were able to develop advanced practice clinician skills to provide treatment independently. The ECHO has also been a place where we can share and learn about newer treatments and approaches in this rapidly-developing field.

## Conclusion

People with chronic HCV currently face fragmented care provision, including diagnosis and cure of chronic HCV. Addiction treatment programs are uniquely situated to either use their existing program infrastructures or expand current programming to increase access to HCV care and treatment. By recognizing the unique, trusted, ongoing clinical role we play in many patients’ lives, we can help their health beyond addiction.

## Abbreviations

CasP: cure as prevention
DAA: direct-acting antiviral
HCV: hepatitis C virus
OUD: opioid use disorder
PWID: people who inject drugs
SVR: sustained virologic response
